# 3,4-Dimethyl-*N*-[(*E*)-3-nitro­benzyl­idene]-1,2-oxazol-5-amine

**DOI:** 10.1107/S160053681003583X

**Published:** 2010-09-11

**Authors:** Abdullah M. Asiri, Salman A. Khan, M. Nawaz Tahir

**Affiliations:** a The Center of Excellence for Advanced Materials Research, King Abdul Aziz University, Jeddah 21589, PO Box 80203, Saudi Arabia; b Department of Chemistry, Faculty of Science, King Abdul Aziz University, Jeddah 21589, PO Box 80203, Saudi Arabia; c Department of Physics, University of Sargodha, Sargodha, Pakistan

## Abstract

In the title compound, C_12_H_11_N_3_O_3_, the dihedral angle between the 3-nitro­benzaldehyde and 5-amino-3,4-dimethyl-1,2-oxazole moieties is 2.46 (12)°. The mol­ecule is close to planar, the r.m.s. deviation for the non-H atoms being 0.028 Å. The packing only features van der Waals inter­actions between the mol­ecules.

## Related literature

For background and related crystal structures, see: Asiri *et al.* (2010**a* ▶,*b* ▶,*c* ▶,d* ▶).
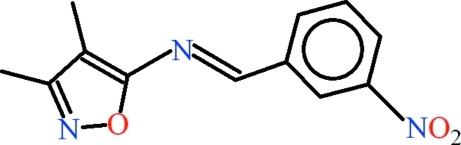


## Experimental

### 

#### Crystal data

C_12_H_11_N_3_O_3_
*M* *_r_* = 245.24
Monoclinic, 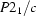

*a* = 12.602 (2) Å
*b* = 3.9267 (6) Å
*c* = 23.366 (4) Å
β = 94.791 (9)°
*V* = 1152.3 (3) Å^3^
*Z* = 4
Mo *K*α radiation
μ = 0.11 mm^−1^
*T* = 296 K
0.22 × 0.08 × 0.06 mm

#### Data collection

Bruker Kappa APEXII CCD diffractometer
Absorption correction: multi-scan (*SADABS*; Bruker, 2005 ▶) *T*_min_ = 0.992, *T*_max_ = 0.995
8616 measured reflections
2046 independent reflections
846 reflections with *I* > 2σ(*I*)
*R*_int_ = 0.097

#### Refinement

*R*[*F* ^2^ > 2σ(*F* ^2^)] = 0.061
*wR*(*F* ^2^) = 0.161
*S* = 0.99
2046 reflections
166 parameters
H-atom parameters constrained
Δρ_max_ = 0.16 e Å^−3^
Δρ_min_ = −0.19 e Å^−3^

### 

Data collection: *APEX2* (Bruker, 2009 ▶); cell refinement: *SAINT* (Bruker, 2009 ▶); data reduction: *SAINT*; program(s) used to solve structure: *SHELXS97* (Sheldrick, 2008 ▶); program(s) used to refine structure: *SHELXL97* (Sheldrick, 2008 ▶); molecular graphics: *ORTEP-3* (Farrugia, 1997 ▶) and *PLATON* (Spek, 2009 ▶); software used to prepare material for publication: *WinGX* (Farrugia, 1999 ▶) and *PLATON*.

## Supplementary Material

Crystal structure: contains datablocks global, I. DOI: 10.1107/S160053681003583X/hb5634sup1.cif

Structure factors: contains datablocks I. DOI: 10.1107/S160053681003583X/hb5634Isup2.hkl

Additional supplementary materials:  crystallographic information; 3D view; checkCIF report

## Figures and Tables

**Fig. 1.**
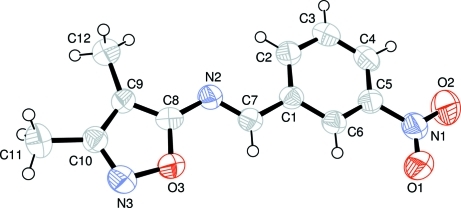
View of (I) with displacement ellipsoids drawn at the 50% probability level.

## supplementary crystallographic information

### Comment

The title compound (I, Fig. 1) is being reported in continuation of our synthetic and structural studies of various Schiff bases of 5-amino-3,4-dimethylisoxazole (Asiri *et al.*, 2010**a*, b, c, d*).

In (I), the 3-nitrobenzaldehyde moiety A (C1—C7/N1/O1/O2) and 5-amino-3,4-dimethylisoxazole moiety B (N2/C8—C12/N3/O3) are planar with r. m. s. deviation of 0.0124 and 0.0099 Å, respectively. The dihedral angle between A/B is 2.46 (12)°. All the heavy atoms (C1—C12/N1—N3/O1—O3) consituate plane with r. m. s. deviation of 0.0276 Å. In this plane, the methyl atom C12 deviates at the maximum with 0.0721 (33) Å. The title compound essentially consists of monomers. There exists no π···π interactions in the crystal.

### Experimental

A mixture of 4-nitrobenzaldehyde (0.33 g, 2.2 mmol) and 5-amino-3,4-dimethylisoxazole (0.24 g, 2.2 mmol) in ethanol (15 ml) was refluxed for 5 h with stirring to give a light yellow precipitate. This material was filtered off and washed with ethanol to give long thin needles of (I).

Yield: 56.45%; m.p. 463–464 K.

IR (KBr) \v_max_ cm^-1^: 3069 (C—H for CH_3_), 2922 (C—H), 1568 (C═C), 1523 (C═N), 1162 (C—N).

### Refinement

The H-atoms were positioned geometrically (C–H = 0.93–0.96 Å) and refined as riding with *U*_iso_(H) = x*U*_eq_(C), where x = 1.5 for methyl and x = 1.2 for other H-atoms.

### Crystal data

**Table:**

C_12_H_11_N_3_O_3_	*F*(000) = 512
*M**_r_* = 245.24	*D*_x_ = 1.414 Mg m^−^^3^
Monoclinic, *P*2_1_/*c*	Mo *K*α radiation, λ = 0.71073 Å
Hall symbol: -P 2ybc	Cell parameters from 846 reflections
*a* = 12.602 (2) Å	θ = 2.3–25.0°
*b* = 3.9267 (6) Å	µ = 0.11 mm^−^^1^
*c* = 23.366 (4) Å	*T* = 296 K
β = 94.791 (9)°	Needle, colorless
*V* = 1152.3 (3) Å^3^	0.22 × 0.08 × 0.06 mm
*Z* = 4	

### Data collection

**Table:**

Bruker Kappa APEXII CCD diffractometer	2046 independent reflections
Radiation source: fine-focus sealed tube	846 reflections with *I* > 2σ(*I*)
graphite	*R*_int_ = 0.097
Detector resolution: 8.20 pixels mm^-1^	θ_max_ = 25.0°, θ_min_ = 2.3°
ω scans	*h* = −15→15
Absorption correction: multi-scan (*SADABS*; Bruker, 2005)	*k* = −4→4
*T*_min_ = 0.992, *T*_max_ = 0.995	*l* = −27→27
8616 measured reflections	

### Refinement

**Table:**

Refinement on *F*^2^	Primary atom site location: structure-invariant direct methods
Least-squares matrix: full	Secondary atom site location: difference Fourier map
*R*[*F*^2^ > 2σ(*F*^2^)] = 0.061	Hydrogen site location: inferred from neighbouring sites
*wR*(*F*^2^) = 0.161	H-atom parameters constrained
*S* = 0.99	*w* = 1/[σ^2^(*F*_o_^2^) + (0.0341*P*)^2^] where *P* = (*F*_o_^2^ + 2*F*_c_^2^)/3
2046 reflections	(Δ/σ)_max_ < 0.001
166 parameters	Δρ_max_ = 0.16 e Å^−^^3^
0 restraints	Δρ_min_ = −0.19 e Å^−^^3^

### Special details

**Table:**

Geometry. Bond distances, angles *etc*. have been calculated using the rounded fractional coordinates. All su's are estimated from the variances of the (full) variance-covariance matrix. The cell e.s.d.'s are taken into account in the estimation of distances, angles and torsion angles
Refinement. Refinement of *F*^2^ against ALL reflections. The weighted *R*-factor *wR* and goodness of fit *S* are based on *F*^2^, conventional *R*-factors *R* are based on *F*, with *F* set to zero for negative *F*^2^. The threshold expression of *F*^2^ > σ(*F*^2^) is used only for calculating *R*-factors(gt) *etc*. and is not relevant to the choice of reflections for refinement. *R*-factors based on *F*^2^ are statistically about twice as large as those based on *F*, and *R*- factors based on ALL data will be even larger.

### Fractional atomic coordinates and isotropic or equivalent isotropic displacement parameters (Å^2^)

**Table:**

	*x*	*y*	*z*	*U*_iso_*/*U*_eq_	
O1	0.0661 (2)	0.9618 (9)	0.11316 (16)	0.0932 (16)	
O2	0.0217 (3)	0.7756 (9)	0.19373 (16)	0.1103 (19)	
O3	0.5961 (2)	0.7628 (6)	0.02581 (12)	0.0630 (11)	
N1	0.0873 (3)	0.8153 (10)	0.15894 (19)	0.0708 (17)	
N2	0.5490 (3)	0.5694 (8)	0.11691 (13)	0.0523 (12)	
N3	0.6882 (3)	0.7702 (8)	−0.00509 (15)	0.0660 (17)	
C1	0.3736 (3)	0.6218 (9)	0.14659 (17)	0.0475 (17)	
C2	0.3985 (3)	0.4605 (10)	0.19866 (18)	0.0579 (17)	
C3	0.3218 (4)	0.4146 (10)	0.23763 (18)	0.0629 (17)	
C4	0.2194 (4)	0.5255 (10)	0.22380 (19)	0.0618 (17)	
C5	0.1954 (3)	0.6856 (10)	0.17248 (19)	0.0534 (17)	
C6	0.2697 (3)	0.7379 (9)	0.13294 (17)	0.0511 (17)	
C7	0.4530 (3)	0.6720 (9)	0.10518 (17)	0.0527 (17)	
C8	0.6232 (3)	0.6142 (10)	0.07766 (17)	0.0521 (17)	
C9	0.7266 (3)	0.5238 (9)	0.08165 (17)	0.0485 (17)	
C10	0.7629 (3)	0.6275 (9)	0.02938 (19)	0.0513 (17)	
C11	0.8723 (3)	0.5881 (10)	0.00968 (19)	0.0727 (19)	
C12	0.7889 (3)	0.3614 (10)	0.13104 (17)	0.0672 (17)	
H2	0.46743	0.38159	0.20778	0.0695*	
H3	0.33974	0.30952	0.27277	0.0758*	
H4	0.16713	0.49195	0.24908	0.0742*	
H6	0.25095	0.84733	0.09826	0.0611*	
H7	0.43386	0.77846	0.07029	0.0629*	
H11A	0.87321	0.67477	−0.02869	0.1088*	
H11B	0.89153	0.35146	0.01024	0.1088*	
H11C	0.92235	0.71279	0.03486	0.1088*	
H12A	0.82773	0.53290	0.15333	0.1006*	
H12B	0.83789	0.20017	0.11712	0.1006*	
H12C	0.74137	0.24627	0.15460	0.1006*	

### Atomic displacement parameters (Å^2^)

**Table:**

	*U*^11^	*U*^22^	*U*^33^	*U*^12^	*U*^13^	*U*^23^
O1	0.064 (2)	0.141 (3)	0.074 (3)	0.0185 (19)	0.003 (2)	0.011 (2)
O2	0.066 (3)	0.170 (4)	0.100 (3)	0.011 (2)	0.037 (2)	0.017 (2)
O3	0.0537 (19)	0.084 (2)	0.051 (2)	0.0055 (14)	0.0022 (15)	0.0092 (15)
N1	0.057 (3)	0.097 (3)	0.059 (3)	0.002 (2)	0.009 (2)	−0.011 (2)
N2	0.048 (2)	0.061 (2)	0.048 (2)	−0.0018 (17)	0.0047 (18)	−0.0012 (16)
N3	0.065 (3)	0.080 (3)	0.054 (3)	−0.001 (2)	0.011 (2)	0.0068 (19)
C1	0.050 (3)	0.047 (3)	0.045 (3)	−0.0016 (19)	0.001 (2)	−0.005 (2)
C2	0.052 (3)	0.068 (3)	0.053 (3)	−0.001 (2)	0.000 (2)	−0.010 (2)
C3	0.073 (3)	0.071 (3)	0.045 (3)	−0.003 (2)	0.006 (3)	−0.003 (2)
C4	0.065 (3)	0.073 (3)	0.049 (3)	−0.007 (2)	0.014 (2)	−0.010 (2)
C5	0.046 (3)	0.059 (3)	0.054 (3)	−0.006 (2)	−0.002 (2)	−0.013 (2)
C6	0.050 (3)	0.058 (3)	0.045 (3)	−0.0016 (19)	0.003 (2)	−0.005 (2)
C7	0.049 (3)	0.065 (3)	0.044 (3)	−0.001 (2)	0.004 (2)	0.000 (2)
C8	0.056 (3)	0.056 (3)	0.043 (3)	−0.001 (2)	−0.003 (2)	0.003 (2)
C9	0.042 (3)	0.055 (3)	0.048 (3)	0.001 (2)	0.001 (2)	−0.003 (2)
C10	0.049 (3)	0.053 (3)	0.052 (3)	0.001 (2)	0.004 (2)	−0.005 (2)
C11	0.063 (3)	0.079 (3)	0.079 (4)	0.000 (2)	0.024 (3)	0.000 (3)
C12	0.064 (3)	0.078 (3)	0.059 (3)	0.010 (2)	0.002 (2)	0.002 (2)

### Geometric parameters (Å, °)

**Table:**

O1—N1	1.224 (6)	C8—C9	1.346 (5)
O2—N1	1.217 (6)	C9—C10	1.400 (6)
O3—N3	1.418 (5)	C9—C12	1.484 (5)
O3—C8	1.362 (5)	C10—C11	1.498 (5)
N1—C5	1.464 (5)	C2—H2	0.9300
N2—C7	1.283 (5)	C3—H3	0.9300
N2—C8	1.375 (5)	C4—H4	0.9300
N3—C10	1.312 (5)	C6—H6	0.9300
C1—C2	1.384 (6)	C7—H7	0.9300
C1—C6	1.398 (5)	C11—H11A	0.9600
C1—C7	1.462 (5)	C11—H11B	0.9600
C2—C3	1.394 (6)	C11—H11C	0.9600
C3—C4	1.375 (7)	C12—H12A	0.9600
C4—C5	1.365 (6)	C12—H12B	0.9600
C5—C6	1.385 (6)	C12—H12C	0.9600
			
N3—O3—C8	107.9 (3)	N3—C10—C11	119.2 (4)
O1—N1—O2	122.2 (4)	C9—C10—C11	127.9 (4)
O1—N1—C5	118.9 (4)	C1—C2—H2	120.00
O2—N1—C5	118.9 (4)	C3—C2—H2	120.00
C7—N2—C8	120.0 (3)	C2—C3—H3	120.00
O3—N3—C10	104.8 (3)	C4—C3—H3	120.00
C2—C1—C6	119.3 (4)	C3—C4—H4	120.00
C2—C1—C7	121.7 (3)	C5—C4—H4	120.00
C6—C1—C7	119.0 (3)	C1—C6—H6	121.00
C1—C2—C3	121.0 (4)	C5—C6—H6	121.00
C2—C3—C4	119.6 (4)	N2—C7—H7	120.00
C3—C4—C5	119.2 (4)	C1—C7—H7	120.00
N1—C5—C4	118.9 (4)	C10—C11—H11A	109.00
N1—C5—C6	118.2 (4)	C10—C11—H11B	109.00
C4—C5—C6	122.8 (4)	C10—C11—H11C	109.00
C1—C6—C5	118.2 (4)	H11A—C11—H11B	109.00
N2—C7—C1	120.1 (3)	H11A—C11—H11C	109.00
O3—C8—N2	120.9 (3)	H11B—C11—H11C	109.00
O3—C8—C9	110.1 (3)	C9—C12—H12A	109.00
N2—C8—C9	128.9 (4)	C9—C12—H12B	109.00
C8—C9—C10	104.4 (3)	C9—C12—H12C	109.00
C8—C9—C12	127.8 (4)	H12A—C12—H12B	109.00
C10—C9—C12	127.8 (3)	H12A—C12—H12C	109.00
N3—C10—C9	112.9 (3)	H12B—C12—H12C	109.00
			
C8—O3—N3—C10	0.2 (4)	C7—C1—C6—C5	179.5 (3)
N3—O3—C8—C9	−0.5 (4)	C2—C1—C7—N2	−0.9 (6)
N3—O3—C8—N2	−179.4 (3)	C1—C2—C3—C4	1.2 (6)
O2—N1—C5—C4	−0.3 (6)	C2—C3—C4—C5	−1.4 (6)
O2—N1—C5—C6	−178.9 (4)	C3—C4—C5—N1	−177.6 (4)
O1—N1—C5—C4	179.3 (4)	C3—C4—C5—C6	0.9 (6)
O1—N1—C5—C6	0.7 (6)	N1—C5—C6—C1	178.4 (3)
C7—N2—C8—C9	−179.6 (4)	C4—C5—C6—C1	−0.2 (6)
C8—N2—C7—C1	179.4 (3)	O3—C8—C9—C10	0.5 (4)
C7—N2—C8—O3	−0.9 (5)	N2—C8—C9—C12	−2.5 (7)
O3—N3—C10—C11	179.1 (3)	O3—C8—C9—C12	178.7 (3)
O3—N3—C10—C9	0.1 (4)	N2—C8—C9—C10	179.4 (4)
C6—C1—C2—C3	−0.4 (6)	C8—C9—C10—N3	−0.4 (4)
C7—C1—C2—C3	−180.0 (4)	C12—C9—C10—C11	2.5 (6)
C6—C1—C7—N2	179.6 (3)	C8—C9—C10—C11	−179.3 (4)
C2—C1—C6—C5	−0.1 (5)	C12—C9—C10—N3	−178.6 (4)
